# Event-Based User Classification in Weibo Media

**DOI:** 10.1155/2014/479872

**Published:** 2014-07-16

**Authors:** Liang Guo, Wendong Wang, Shiduan Cheng, Xirong Que

**Affiliations:** State Key Laboratory of Networking and Switching Technology, Beijing University of Posts and Telecommunications, Beijing 100876, China

## Abstract

Weibo media, known as the real-time microblogging services, has attracted massive attention and support from social network users. Weibo platform offers an opportunity for people to access information and changes the way people acquire and disseminate information significantly. Meanwhile, it enables people to respond to the social events in a more convenient way. Much of the information in Weibo media is related to some events. Users who post different contents, and exert different behavior or attitude may lead to different contribution to the specific event. Therefore, classifying the large amount of uncategorized social circles generated in Weibo media automatically from the perspective of events has been a promising task. Under this circumstance, in order to effectively organize and manage the huge amounts of users, thereby further managing their contents, we address the task of user classification in a more granular, event-based approach in this paper. By analyzing real data collected from Sina Weibo, we investigate the Weibo properties and utilize both content information and social network information to classify the numerous users into four primary groups: celebrities, organizations/media accounts, grassroots stars, and ordinary individuals. The experiments results show that our method identifies the user categories accurately.

## 1. Introduction

The real-time microblogging services [1], called Weibo media in China, have surged in popularity in recent years. As an information publishing and sharing platform, Weibo media enables people to garner, publish, and disseminate information, while enjoying the fun of sharing and socializing simultaneously. Sina Weibo (http://www.weibo.com/), provided by SINA Corporation in 2009, is the most popular microblogging service in China. It demonstrates some different characteristics from Twitter in functionality. For instance, Sina Weibo supports threaded comments feature, groups, audios, messages, direct videos uploading and sharing, and so forth. It is much more akin to a hybrid of Twitter and Facebook, which combines the information sharing and social interaction perfectly. With Sina Weibo platform, more and more people gather together spontaneously to involve in certain discussions around social events. Consequently, in response to these events, substantial amounts of user contents have been generated and spread, accompanied with the formation of ever-increasing social circles. In this context, a problem of significant interest is to classify these cluttered social circles and manage the huge volume of information from the growing and evolving set of unstructured data generated in Sina Weibo.

There have been some researches dedicated to clustering the dispersed users with similar interests into the same categories [2, 3] or classifying all the users a single user follows to discover user's social circles and provide a clearer picture of his/her interests [4]. There are still other works focusing on categorizing user posts to help organize the incoming Weibo feed [5]. The ultimate goal of these conventional classification tasks is to recommend potential interesting users/topics to the appropriate audiences [6]. Some work has shown that classification-based similarity metrics with machine learning significantly outperform other similarity approaches in different cold-start situations under different degrees of data sparseness [7]. However, these prior efforts are mainly network-wide user classification. Few of them consider the fact that, around a particular event, the premise is that these users have similar interests in that event. Users associated with the same event may exhibit different roles or characteristics and in turn lead to different contribution to the developing trends of the event. Therefore, there is a need to reclassify the numerous users automatically from the perspective of events. In this paper, we cast the problem as an event-based user classification task, which explores the user categories in a more fine-grained method. By learning and analyzing the vocabulary usage features and interactive features of users, a framework for event specific user classification in Weibo media is proposed. Around a social event, we try to capture the related user and network characteristics to differentiate the Weibo users into four primary categories: celebrities, organizations/media accounts, grassroots stars, and ordinary individuals, and thus reflect the different concerns taken by the relevant groups. The definition of the four user categories is given below.

1. Celebrities are defined as the domain experts and famous persons from all walks of life, who have passed the official “V” identity authentication of Sina Weibo (The “V” authentication of Sina Weibo will be introduced in Section 3.1). This category includes elite users, movie stars, professional athletes, and expert users. The celebrities usually have wide public attention and high influence on others.
2. Organizations/media accounts are entities which represented some social, political, business, or media agency. It is used for marketing, public relations, or customer services. The organizations/media accounts also have the official “V” certification of Sina Weibo.
3. Grassroots stars are a special user group in Weibo media. It is registered by the nonofficial, unauthenticated users who maintain the Weibo account to reflect their professional interests. Compared with the celebrities, the grassroots stars only provide theme-related contents and almost never post about their own life. On the contrary, the celebrities concern about varied areas. Even just releasing their personal feelings, the celebrities' posts will still cause a large amount of reply and forwarding. Although the grassroots stars do not have the famous genes at first, they can attract more and more attentions because of the fresh, unique, or original contents.
4. Ordinary individuals are simply active Weibo users who do not belong to the above three categories. Ordinary individuals post updates about their daily lives, contact with social friends, and visit Weibo frequently to share interesting information.

We take the emergency event “Ya'an Earthquake” as an example to illustrate how users behave from different circles on a particular event. Some ordinary individuals may release the first-hand facts or eyewitness information. The celebrities may help to redistribute the fact of the current progress to a larger audiences and concern about the donation and disaster recovery. The organizations and media accounts devote to coordinate rescue efforts for the emergency, while most of the other ordinary individuals forward the facts published by the formers. As is expected, the ordinary individuals tend to use more blessing words in their contents. In this event, the ordinary individuals who publish the original first-hand facts or eyewitness information might attract large number of attention and become grassroots stars eventually. Meanwhile, the keywords contained in users' posts may also be different. Therefore, text analysis method on Weibo contents and the social relation analysis method on Weibo network are both utilized, while the Naïve Bayes classification method implemented in Data Mining tool Weka [8] is employed to realize the classification of Weibo users in this paper. This is the first work to classify the Sina Weibo users from the events' perspective. The contribution of this paper is as follows.

1. We introduce a method for classifying users from the perspective of social events in Weibo media. The primary user categories are classified as celebrities, organizations/media accounts, grassroots stars, and ordinary individuals.
2. We find that users from different categories emphasize different aspects against the same events, expressed as the different vocabulary usage and interaction behaviors.
3. We provide an in-depth analysis of the experiment results, revealing that making use of user's profile features, interaction features, content features, and the social network features together can be of great benefit to exploiting the user characteristics. Furthermore, URLs can be used effectively to classify the user categories, especially to distinguish organizations/media accounts from the others.
4. The event-based scenario provides finer granularity in user classification tasks. It can also be helpful in multiple applications, such as user recommendation, topic recommendation, and influence of user extraction.
5. Although the classification task of this paper is based on Sina Weibo, the event-based classification method is also applicable to other social medias, such as Twitter, whose feature is the subset of Sina Weibo, or other platforms which have the need to address the user classification problem with respect to events.

The remainder of this paper is organized as follows. In the next section, we review the previous work on user classification. In Section 3, an overview of the user classification model is introduced. The concrete approach of the classification model is presented in Section 4.  Section 5 outlines our experiment results on the datasets of Sina Weibo. Finally, we conclude this paper and state several directions for future work in Section 6.

## 2. Related Work

With the proliferation in popularity of social networking services, the subject of social media in general and microblogging services in particular has attracted considerable ongoing research works. As mentioned in the above section, a number of previous works have explored the research on user classification in microblogging services, mostly in Twitter services.

Java et al. [1] analyze the microblogging phenomena by studying the topological and geographical properties of Twitter's social network. They find that users talk about their daily activities and seek or share information using Twitter. In addition, they utilize Clique Percolation Method (CPM) to find the user communities. Wu et al. [9] use Twitter lists to distinguish between elite users and ordinary users. The elite users include celebrities, bloggers, and representatives of media outlets and other formal organizations. They conclude that 50% of URLs are generated by 20 K elite users. This paper also indicates that users within the same categories show some level of homophily.

There are also some works focusing on analyzing the specific user groups in Twitter, such as the classification of political affiliation [10], ethnicity, and affinity for a particular business. Pennacchiotti et al. classify Twitter users into three types using machine learning approach, namely, political affiliation (Democrats and Republicans), ethnicity, and Starbucks fans [2, 3]. The main contribution of their works is to build a general, scalable, and robust architecture for automatically computing the values of given user attributes for a large set of Twitter users and fulfill the task of user classification sequentially. Bergsma et al. [11] propose an algorithm to cluster the observed attributes of hundreds of millions of Twitter users. The efficacy of these clusters is then evaluated on a diverse set of classification tasks that predict hidden users properties, such as ethnicity, gender, and location. Gayo-Avello [12] studies the task of classification of demographic attributes and proposes a semisupervised algorithm to perform user profiling in social networks, which enables to label unknown users from small partially labeled samples. Furthermore, the measures to minimize the privacy leaks while conducting the social graph Data Mining process are outlined in his work. Zhao et al. [13] focus on classifying users' sentiment trends on topic level, including positive, negative, and neutral. McAuley and Leskovec [4] try to automatically identify user's personal social circles instead of categorizing the huge amount of users from the complete social network into several groups. They cast the problem as to find communities or clusters in one user's ego network. On the grounds of the diverse evaluation dataset from Facebook, Google+, and Twitter, the experiments reveal that social circles can be accurately detected using a combination of both network and profile information. In [14], the authors try to classify the followers in Twitter by analyzing followers' retweet information, profile, and recent tweet sentiment information. Li and Zhang [15] focus on the task of short text classification and user interest tagging. They propose a method utilizing a semisupervised coupled mutual reinforcement framework based on social correlation to simultaneously classify short text and tag user interest.

The most relevant work with our research is [16] which builds an automatic classifier for user types on Twitter, focusing on three core user categories that are reflective of the information production and consumption processes around events: organizations, journalists/bloggers, and ordinary individuals. Our paper differs from this earlier work by shifting attention from the perspective of information production and consumption in Twitter to the interests or behavior habits of different user groups exhibited in Chinese Weibo media around the events. As noted above, the Chinese Weibo is quite different from Twitter in linguistic features and network structure features. Kwak et al. [17] analyze the structure of Twitter and its difference from Weibo. They use different methods to identify the influentials in Twitter while the tweets of trending topics and tweets' diffusion are analyzed afterwards. Finally, they conclude from the highly skewed nature of the distribution of followers and the low rate of reciprocated ties that Twitter resembles an information sharing network more closely than a social network.

Therefore, our work confirms the issue by identifying specific categories in terms of the events spread in Weibo media, whose features are different in comparison to Twitter. In addition to the application of recommending interesting users or topics for target users, our work can also help users organize their incoming contents and filter the Weibo content to present the appropriate information to relevant users around a specific event.

## 3. Model Overview

**Figure 1:**
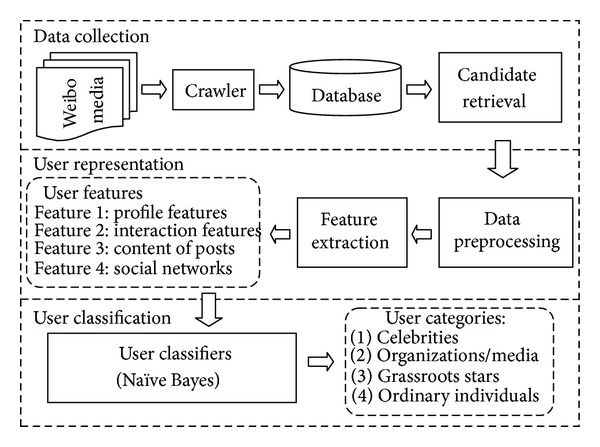
Event-based user classification analysis framework.

As noted above, we focus on four user types: celebrities, organizations/media accounts, grassroots stars, and ordinary individuals. An overview of the event-based user classification analysis framework is outlined in Figure 1. The framework consists of three modules, which are data collection module, user representation module, and user classification module. Before we depict each module in detail, there is some preliminary knowledge about Sina Weibo needed to be explained first which will be referenced in the remainder of this paper.

### 3.1. Features of Sina Weibo

1. *Post*. Post represents the content of the microblogging status up to 140 characters in length, which supports multiple information formats, such as the uploaded video, voice, and pictures.
2. *Following*. Following is the basic behavior of Weibo users, which aims to make other people's posts appear in users' own timeline. When A follows B, it indicates that A is a follower of B and B is a friend of A.
3. *#HashName#*. Users can add hashtags in their posts with the #HashName# format. By using hashtags, the posts are labeled with a specific topic or event.
4. *@UserName*. This is a format to mention or talk to other people. Multiple users can be addressed in a single post. The format can also be considered as a redirect method to lead the posts to the particular users.
5. *Reply*. Sina Weibo provides the function of replying, which empowers users to respond to and discuss the posts. It indicates a level of interest, support, or opposition on the posts and also promotes the social interaction between Weibo users.
6. *Repost*. Repost, with the “//@UserName” format, is quite similar to Twitter's retweet function. It is a repeat of another user's post. The purpose of this function is to redistribute the post on one's own feed in the hopes of broadcasting it to a larger audience, which also demonstrates some degree of interest, support, or concern of the contents.
7. *V*. Some users who have passed the official “V” authentication could obtain a “V” label next to their username. This label represents a real and certified identity and also stands for a higher degree of credibility and influence of the user account.

### 3.2. Problem Definition

In this paper, we cast the problem as an event-specific user classification task. The definition of “event” we use is the same as the topic detection and tracking (TDT) event detection task from [18]: an event identifies something (nontrivial) happening in a certain place at a certain time. Weibo media is a good platform for discussing and diffusing hot topics. We treat the popular hot topics spread in Sina Weibo as the events we research on. This conforms to the definition of “event” proposed in [18].

According to the homophily phenomenon in social networks, similar users are inclined to gather together to discuss the event of the same interest.  Table 1 lists the keywords used by different categories of users according to the same event “The death of Margaret Thatcher.” This is the hot topic in Weibo on April 8, 2013, and the next few days. From the table, we can garner some information that people from different categories emphasize different types of content on the same event, resulting in different keywords appearing in their posts. Hence, the crux of our work is to capture the different aspects of the same event that different users pay attention to.

Given a set of Weibo users associated with an event, the problem that we address in this paper is to classify this set of users into groups such that each group corresponds to all users that are associated with a certain category (e.g., celebrities, organizations/media accounts, grassroots stars, and ordinary individuals) and then to analyze the specific behavior of the different categories of users.

*Assumption*. For each event, we assume that the same user can only fall into one category for simplicity.

## 4. Approach

In this section, we will introduce the specific approach of user classification, while providing the detail description of the analysis framework presented in Figure 1.

### 4.1. Data Collection Module

First, we collect the dataset from the Chinese biggest Weibo site, Sina Weibo. The candidate retrieval process of data collection module consists of three steps.

1. *Events Selection*. Since the focus of this paper is to classify user categories spanning different events, the events selection process will be simplified from automatic to manual identification. Even so, we try to cover different aspects as much as possible to make our conclusion not biased to some domain specific events.
2. *Posts Retrieval*. According to the collected events, the posts retrieval step relies on a set of SQL queries that build on the events. The corresponding posts containing the keywords of the event are fetched for further content analysis.
3. *Users Retrieval*. We glean users' information in accordance with the set of posts in this step. For each user, the required information is gathered to construct the user features, including user profile and user's interaction information.

### 4.2. User Representation Module

Extracting user characteristics constitutes an important step towards user classification. Therefore, we cast the problem in this module as the feature extraction and representation task. However, the collected candidate data must be preprocessed first so that it can be used for the feature extraction process. Afterwards, we propose a set of features for characterizing Weibo users, including both content and network metrics.

In Sina Weibo, there exist two types of important information, namely, user information and the social relation information, which will be utilized in this paper to capture the features of user classification. User information includes the user's profile, the messages they post, the social features they possess (followers and friends), and their social interaction with others (@, repost, reply, etc.). The social relation information is used as a supplementary method to enhance the user characteristics. The four major features extracted in our classification model are listed as follows.

1. * Profile Features*. From the dataset, we garner a lot of information about user's profile, while reserving the useful ones in terms of a vector.
  - *Num.followers*. The number of user's followers indicates the user's popularity in some ways.
  - *Num.friends*. It is the number of user's friends.
  - *Location*. Users may fill out the location information while registering their Weibo account.
  - *Description*. User's description is a free text, which can be used to collect user's interests or preferences.
  - *V*. There are only two possible values {0, 1} of this property. Users marked with a *V* label get a value of 1, otherwise 0.
  - *Date_account_creation*. The creation date of a user account indicates user's microblogging age.

Overall, a vector describing user's profile feature is shown as follows:

(1) $$\begin{array}{c} \vec{PF\left(u\right)}=\left(\begin{array}{c} \text{Num}.\text{followers}\\ \text{Num}.\text{friends}\\ \text{Location}\\ \text{Description}\\ \text{V}\left\{0,1\right\}\\ \text{Date}\_\text{account}\_\text{creation} \end{array}\right). \end{array}$$

(2) * Interaction Features*. The user category could be identified by studying users' activity characteristics. For instance, we can look at how much time a user spends one day in Sina Weibo, how the user interacts with others, and the actions user performs. To describe these behaviors in mathematical manner, we extract several characteristics in terms of a vector as the interaction features, which will be useful for identifying the user categories. The specific features are as follows:
  - IF_1_: number of posts published by the user;
  - IF_2_: number and fraction of posts that are reposted by other users;
  - IF_3_: number and fraction of posts that are replied by other users;
  - IF_4_: average number of #HashName# per post;
  - IF_5_: average time and standard deviation between posts;
  - IF_6_: average number and standard deviation of posts per day.

Therefore, the interaction features are represented by these measures as follows:

(2) $$\begin{array}{c} \vec{\text{IF}\left(u\right)}=\left(\text{I}{\text{F}}_{1},\text{I}{\text{F}}_{2},\text{I}{\text{F}}_{3},\text{I}{\text{F}}_{4},\text{I}{\text{F}}_{5},\text{I}{\text{F}}_{6}\right). \end{array}$$

(3) * Content of Posts*. The content of posts indicates the users' interests and their usage habit of vocabulary and lexical expression. For example, ordinary individuals tend to use the trendy words more than the celebrities. Grassroots stars tend to publish or repost the ignored social events concerning the social livelihood of the people more than the ordinary individuals. Meanwhile, for the same event, people's concerns are not the same, which can be reflected in their Weibo content. Therefore, we focus on analyzing the characteristics explicitly or implicitly contained in user's posts, to prepare for the user classification task.

We use three kinds of relations to describe the content feature, which are contents-users relationship, contents-categories relationship, and users-categories relationship. Given an event, we try to infer the users-categories relationship by calculating and using the contents-users and the contents-categories relationships. The content feature extraction procedure is divided into three steps.

In the first step, the contents-users relationship is quantitatively described by considering the degree of preference for a user using word *w*_*i*_. Equation (3) indicates the preference probability of user *u* to use a particular word, which expresses the user's habit of using the word *w*_*i*_. The degree of preference Preference(*u*, *w*_*i*_) is defined as the usage frequency of user *u* to use the word *w*_*i*_. *Wu* denotes all the words that user *u* has ever used:

(3) $$\begin{array}{c} P_{u}\left(w_{i}\right)=\frac{\text{Preference}\left(u,w_{i}\right)}{\sum_{w\in Wu}\text{Preference}\left(u,w\right)}. \end{array}$$

In the second step, the contents-categories relationship is captured from

(4) $$\begin{array}{c} P\left(c_{i}\;|\;w_{j}\right)=\frac{\sum_{s_{i}\in c_{i}}\text{Preference}\left(s_{i},w_{j}\right)}{\sum_{k=1}^{n}\sum_{s_{k}\in c_{k}}\text{Preference}\left(s_{k},w_{j}\right)}, \end{array}$$

where *n* = 4 and *c*_*i*_ ∈ {*c*_1_ = celebrity, *c*_2_ = Org/Media, *c*_3_ = Grassroots, *c*_4_ = Ordinary} in this paper. Equation (4) represents the probability that word *w*_*j*_ belongs to category *c*_*i*_. *s*_*i*_ indicates the seed users falling into category *c*_*i*_. In (4), the numerator stands for the summation of the degree of preference for all the seed users in category *c*_*i*_ using word *w*_*j*_, while the denominator summarizes the degree of preference of all the seed users from the whole four categories on word *w*_*j*_.

Finally, in the third step, the users-categories relationship is depicted in

(5) $$\begin{array}{c} P\left(u\in c_{i}\right)=\frac{\sum_{w\in Wc_{i}}\text{Preference}\left(u,w\right)}{\sum_{w^{\prime }\in Wu}\text{Preference}\left(u,w^{\prime }\right)}. \end{array}$$

Given a user *u*, (5) captures the probability that *u* belongs to category *c*_*i*_, where *Wc*_*i*_ represents the set of words used by the users in the category *c*_*i*_.

(4) * Social Networks*. In addition to user generated contents, the social connections established by Weibo users could also be utilized as a measure to identify the social circles user belongs to. In this paper, we model the social relation strength from user *u*_*i*_ to *u*_*j*_ as *R*(*u*_*i*_, *u*_*j*_) in (6), while considering the degree of preference that user *u*_*i*_ gives to *u*_*j*_. The degree of preference Preference(*u*_*i*_, *u*_*j*_) indicates the behavior frequency that *u*_*i*_ points to *u*_*j*_, such as @, repost, or reply. It is assumed here that *u*_*i*_ follows *u*_*j*_, which means that *u*_*i*_ is a follower of *u*_*j*_ and *u*_*j*_ is a friend of *u*_*i*_. Obviously, *R*(*u*_*i*_, *u*_*j*_) ≠ *R*(*u*_*j*_, *u*_*i*_), because of the asymmetrical interpersonal relationship in Sina Weibo:
  (6) $$\begin{array}{c} R\left(u_{i},u_{j}\right)=\frac{\text{Preference}\left(u_{i},u_{j}\right)}{\sum_{k\in F_{i}}\text{Preference}\left(u_{i},u_{k}\right)}, \end{array}$$
  where *F*_*i*_ stands for the friends set of user *u*_*i*_. Hence, a simplified social influence (SI) measure of user *u*_*j*_ is defined in
  (7) $$\begin{array}{c} \text{SI}\left(u_{j}\right)=\frac{1}{\left|\text{Fo}{\text{l}}_{j}\right|}\underset{l\in \text{Fo}{\text{l}}_{j}}{\sum }R\left(u_{l},u_{j}\right), \end{array}$$
  where Fol is the abbreviation of followers and Fol_*j*_ indicates the followers set of *u*_*j*_.

### 4.3. User Classification Module

This component takes as input a small training set of labeled examples and learns a classification model which can then be used to label the large set of Weibo users. Owing to the existing mature classification methods, various classical approaches could be used as classifiers to fulfill our classification task. In this paper, we use Naïve Bayes as the classification method to classify the user categories in Sina Weibo. To learn the classification model, each user is represented as a vector according to the four major features presented in Section 4.2. According to the naïve independence assumptions between the features, given a user *u*_*i*_ with the feature variables *F*(*u*_*i*_) = (*f*_1_^*i*^, *f*_2_^*i*^,…, *f*_*n*_^*i*^), our goal is to classify user *u*_*i*_ into appropriate user category *c*_*j*_, as shown in

(8) classify(f1i,f2i,…,fni) =argmaxcj  P(C=cj)∏k=1nP(fkiC=cj).

From Figure 1, we can see that the output of User Classifier component is the users who are each marked with a Category Label. The specific implementation strategy will be explained in detail in Section 5.

## 5. Experiments

### 5.1. Model Training

To train the classification model, we first gather a set of seed users as the training dataset, who are labeled with one of the four categories. The users categorized as celebrities are obtained from Sina Weibo's “Hall of Fame” application. The “Hall of Fame” page (http://data.weibo.com/top/influence/famous?type=day) lists the top 100 celebrities each day. We gather a random sample of 1000 celebrity users from the 30 days of “Hall of Fame” lists. Similarly, we use the media list (http://data.weibo.com/top/influence/media?type=day) and the government list (http://data.weibo.com/top/influence/govern?type=day) to collect a list of 1500 authorized labeled organizations/media accounts. Besides, we randomly select 1000 users who have more than 4000 followers and do not pass the “V” authentication as the grassroots stars and 1000 users whose followers' number is less than 1000 as the ordinary individuals.

According to the different categories of users described above, we have obtained 4,500 labeled users as the training dataset to learn the classification model. In this paper, we use the Naïve Bayes classification method implemented in open source Data Mining tool Weka with default settings to validate and explore the classification performance. We use 5-fold cross-validation to validate our training model. The confusion matrix is given in Table 2, while Table 3 lists the Precision, Recall, and AUC (ROC Area) results for each user category. The high values of these three metrics indicate that our model can be used to label the Weibo users.

### 5.2. Datasets Description

We conduct a set of experiments over the events spread in Weibo to investigate the effectiveness of the proposed approach. The datasets derived from Sina Weibo site span from September 2009 to July 2013. It consists of 31,516,095 users and 59,839,444 posts. To perform our experiments, we sample four of the most popular Weibo events in 2012 including “Earthquake,” “College Entrance Examination,” “Crack Down Human Traffickers,” “Gangnam Style”, and four other events, which are “Xiaomi Phone,” “2013 Spring Festival,” “Data Mining,” and “Tianyi Li.” These events cover multiple fields, involving news, business, technology, entertainment, and festival, which ensure that our experiments are not biased towards a certain type of event.

Table 4 lists the detail data statistics of each event, including the number of posts, the number of users, and the time intervals of the collected posts. The time span of “2013 Spring Festival” expresses the Chinese traditional festival from New Year's Eve to Lantern Festival.

### 5.3. Data Preprocessing

According to the event related posts, the objective of this process is to extract the major words list associated with the event. In order to avoid using the unrepresentative words in the decision-making of classification process, the text processing method is utilized to filter the text characteristics. Compared with English, there is no obvious delimiter in Chinese text to distinguish the word boundary. Therefore, we must first perform the Chinese word segmentation task. In this paper, we employ NLPIR (http://ictclas.nlpir.org/) as our segmentation tool. NLPIR, also called ICTCLAS, is a Chinese lexical analysis system which supports the function of Chinese word segmentation, POS tagging, and name entity recognition, and so forth. Afterwards, the 1208 Chinese stop words and other non-Chinese noise words are filtered out in order to ensure the validity of the remaining words.

### 5.4. Evaluation Metrics

In order to evaluate our methods, we build the gold standard by manually labeling a cluster of users for each category in an event. The gold standard dataset is labeled from a random sample of users who belongs to the specific event. We have three volunteer testers to label each user into one of the four user categories, but remove the users when there is no agreement between the three testers. Finally, for each of the four user groups, 100 labeled users are adopted to evaluate our classification accuracy.

In this paper, we report our results over three criterions, which are Precision, Recall, and* F*-measure. We use the profile features and the interaction features presented in Section 4 as our baseline, which means the User Classifier component trained only on the profile features and the interaction features. Finally, the Naïve Bayes implemented in Weka tool is employed to realize the classification task and evaluate the validity of the proposed methods in terms of Precision, Recall, and* F*-measure.

### 5.5. Experimental Results

**Figure 2:**
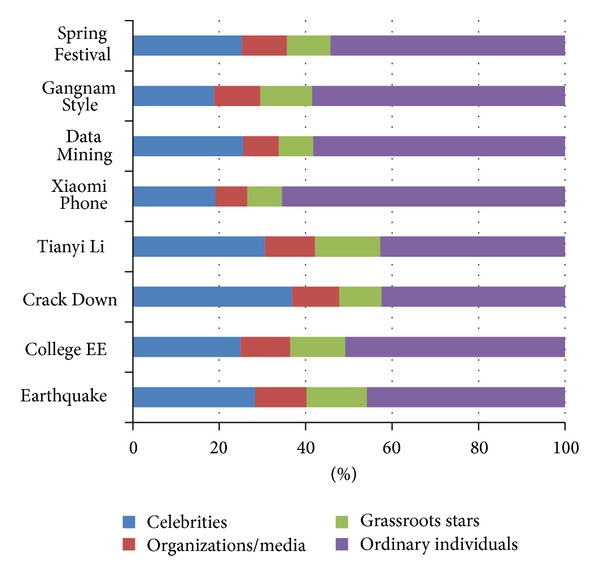
User size distribution on each event.

**Figure 3:**
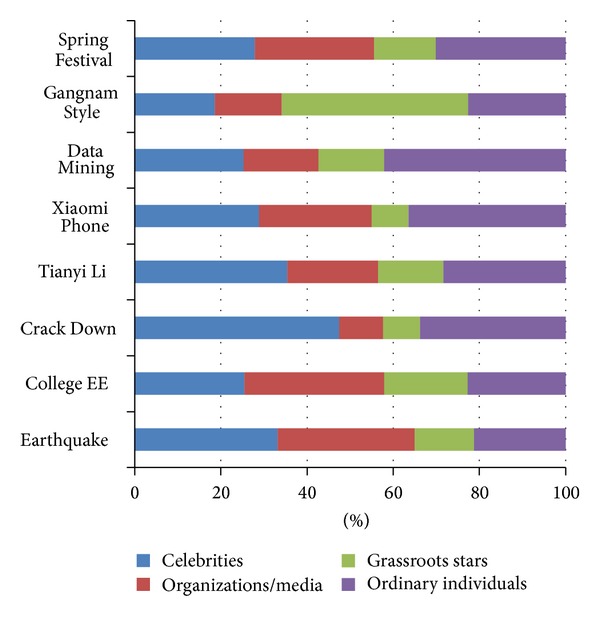
Posts size distribution on each event.

(1) * User Participation Analysis*. We begin with the task of analyzing the user distribution on different events. For each event, the user size proportion of each category is presented in Figure 2, while Figure 3 illustrates the posts distribution from different user categories. This is the preliminary description of the phenomena that different groups of users concern about different events, exerting different levels of participation. As can be seen from the figures, the ordinary individuals occupy a large proportion of user size generally, but the number of posts they update is not the most. Compared with organizations/media accounts and grassroots stars, more of the celebrities participate in discussing the events. However, the posts distribution from these different categories differs with the diverse events. Take the event of “Gangnam Style” as an example; the percentage of grassroots stars is 11.97%, while their posts percentage is up to 43.27%.
(2) * Content Characteristic Analysis*. From Tables 5, 6, 7, 8, 9, 10, 11, and 12, the specific keywords distribution on the eight Weibo events used in this paper are provided. All of the Chinese words have been translated into English. For each event, the top unclassified Cooccurrence Keywords are listed, while the category relevant top contribution keywords are presented too. The contribution degree of a keyword in category *c*_*i*_ is measured in (9), which is expressed by the degree of preference of word *w* from all of the users belonging to that category. We use this definition to plot the distribution of keywords across different user categories:
  (9) $$\begin{array}{c} \text{Contr}{\text{i}}_{c_{i}}\left(w\right)=\underset{u\in c_{i}}{\sum }\text{Preference}\left(u,w\right). \end{array}$$

From these tables, we can differentiate the word using habits of the different categories of users. Users focus on different aspects of the same event by using different keywords in their posts. Meanwhile, from the diversity of the category-specific keywords, we can conclude that the content features of users' Weibo posts are an important factor towards user classification. We choose the event of “Crack Down” as an example; celebrities tend to use the words such as “proposal,” “fund,” and “donation” to show concern about the initiative of cracking down the human traffickers, the establishment of the fund, and the donation matters. The organizations/media accounts emphasize on the action that was taken to combat the trafficking crimes, as well as the coverage of the event. The name of “Shiqu Chen,” who is the director of the cracking down office of Chinese ministry of public security, appears in their posts frequently. The grassroots stars respond to Charles Xue's appeal by participating in the “crack down” campaign and encouraging netizens to photograph the abducted children and spread these children's pictures to rescue them. Finally, the ordinary individuals tend to express their personal feelings on the events, such as support for the antitrafficking action, and sympathy for the abducted children.

**Figure 4:**
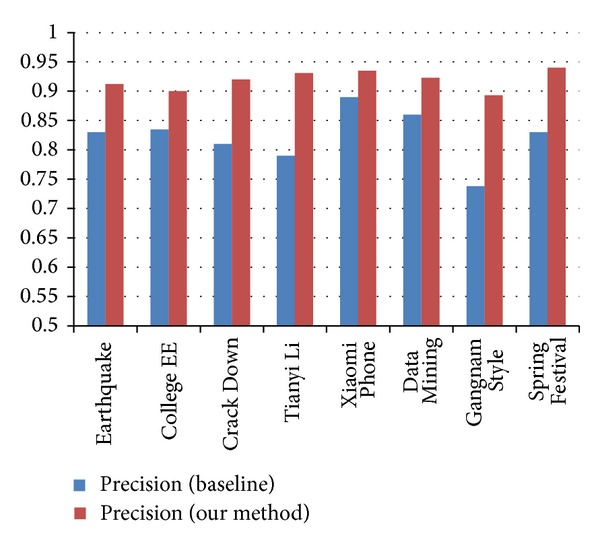
Comparison of Precision measure between baseline and our methods.

**Figure 5:**
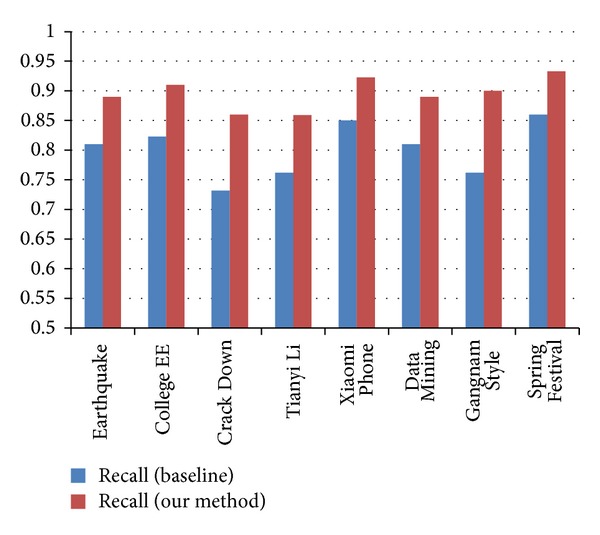
Comparison of Recall measure between baseline and our methods.

**Figure 6:**
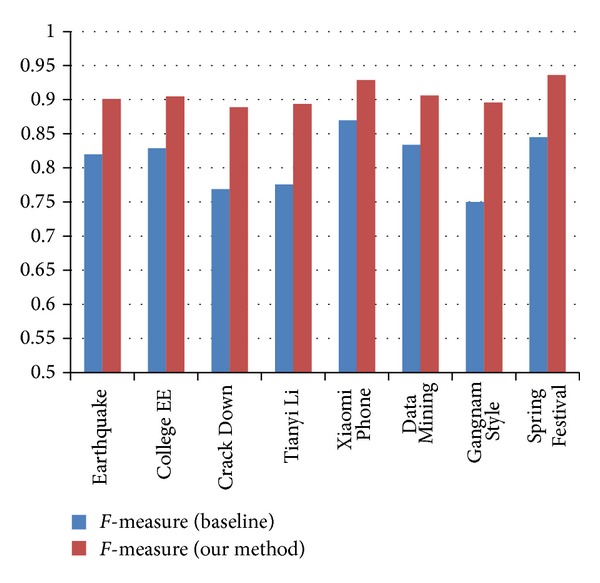
Comparison of* F*-measure between baseline and our methods.

(3) * Classification Accuracy Analysis*. The comparison results of classification accuracy in terms of Precision, Recall, and* F*-measure are outlined in Figures 4, 5, and 6. We observe that our methods get better performance than the baseline. The results significantly explain the necessity of combing the different features together. With the employment of Weibo content information and Weibo network information, the accuracy of the event-based user classification model is improved.
(4) * URLs Usage Analysis*. The absolute quantity of URLs adopted by different categories of users is presented in Table 13. These numbers indicate that generally the posts published by organizations/media accounts contain more URLs information. From Table 13, we can observe that the “Crack Down” and “Tianyi Li” events involve less URLs than the others, because of the small number of relevant posts. Besides, the ordinary individuals publish more URLs than the organizations/media accounts on the event of “Spring Festival,” perhaps due to the convenience of utilizing the network resources to send their blessings and the strong desire of participating in the promotional activities initiated by the business platforms. In conclusion, analyzing the URLs usage condition in Weibo posts is meaningful for identifying the user categories.

## 6. Conclusion and Future Works

In this paper, we present an event-based user classification model in Weibo media. By analyzing the real-world data collected from Sina Weibo site in China, we categorize the cluttered Weibo users into four primary groups: celebrities, organizations/media accounts, grassroots stars, and ordinary individuals from the perspective of different events. Both the Weibo content information and the Weibo network information are utilized to construct user features for the classification model. Meanwhile, we analyze the different users' behaviors and demonstrate that users from different categories show different aspects and their degree of interest in the same event. Therefore, classifying users in event granularity is meaningful both for Weibo platform in organizing and managing the users and the contents and for the Weibo users in categorizing their social circles and the incoming feeds. In particular, the experiment results demonstrate the effectiveness of our methods.

However, there are still some optimization opportunities to explore in our work. First, we do not consider the user overlap between four different user categories in this paper. The multilabel machine learning method will be used in our future research to explore the users with multiple labels. Second, it is a promising work to identify the different possible user groups for an event or a set of events with similar features. Hence, future efforts should focus on analyzing different user groups with respect to similar events. Third, the sentiment factor should be taken into consideration while designing the user classification model. Finally, for a particular event, the users' degree of authority could be different, even if they belong to the same category. Therefore, the event-based authority user extraction and ranking in social media are worth doing further analysis for future research.

## Figures and Tables

**Table 1:** Category-specific keywords on the event of “The death of Margaret Thatcher.”

Category	Category-specific keywords
Category 1	*Biography, China, and *“*Chinese, New Ideas*”

Category 2	*Phoenix Weekly, Former British Prime Minister, Iron Lady, and Today in History *

Category 3	*Grandpa Deng, Oscar movies*, 〈The Iron Lady〉,* Meryl Streep, Worship, and Idol *

Category 4	*Female, Conservative Party, Longest-serving, Mourning, and Death *

**Table 2:** Confusion matrix on 5-fold cross-validation.

True\Predicted	Celebrity	Org./media	Grassroots	Ordinary
Celebrity	912	74	11	3
Org./media	79	1387	28	6
Grassroots	47	23	895	35
Ordinary	11	4	34	951

**Table 3:** Precision, Recall, and AUC results for each user category.

Category	Precision	Recall	AUC (ROC Area)
Celebrity	0.869	0.912	0.901
Org./media	0.932	0.925	0.921
Grassroots	0.925	0.895	0.897
Ordinary	0.956	0.951	0.935

**Table 4:** Data statistics of each event.

Events	#Posts	#Users	Time intervals
#Earthquake#	32992	6586	2012-01-01~2012-12-31
#College Entrance Examination#	43072	8060	2012-01-01~2012-12-31
#Crack Down Human Traffickers#	2610	1152	2012-01-01~2012-12-31
#Tianyi Li#	3322	1412	2010-01-30~2013-07-01
#Xiaomi Phone#	48332	6961	2010-06-04~2013-07-01
#Data Mining#	18693	3699	2009-09-08~2013-07-01
#Gangnam Style#	58546	9312	2012-07-30~2013-07-01
#2013 Spring Festival#	27706	8903	2013-02-09~2013-02-24

**Table 5:** Keywords distribution on the event of “Earthquake.”

Event 1: Earthquake		
Category	Cooccurrence Keywords	Keywords with Category Label
Celebrity	*Earthquake, magnitude, China, hypocenter, depth, stricken area, country, and reports *	*Reconstruction, disaster, victims, influence, donation, and compatriots *
Organizations/media accounts		*Seismological bureau, warning, aftershock, casualties, government, relief, supplies, volunteer, and medical team *
Grassroots stars		*Candle, rest in peace, strong, seisesthesia, aftershock, forward, expert, netizen *
Ordinary individuals		*Pray, Wenchuan, Tangshan, hope, children, hold on, and donate *

**Table 6:** Keywords distribution on the event of “College Entrance Examination.”

Event 2: College Entrance Examination		
Category	Cooccurrence Keywords	Keywords with Category Label
Celebrity	*College Entrance Examination*, *high school*, *candidates*, *parents*, *results*, *admission*, and *enrollment *	*Education, China, fairness, university, and fighting *
Organizations/media accounts		*Countdown, 2012, threshold scores, Ministry of Education, and top scorers *
Grassroots stars		*Reform, sign up, major, household register, composition, and English *
Ordinary individuals		*Tutorship, dream, sprint, review, scores, and voluntary *

**Table 7:** Keywords distribution on the event of “Crack Down.”

Event 3: Crack Down		
Category	Cooccurrence Keywords	Keywords with Category Label
Celebrity	*Trafficking*, *MicroBlog*, *children*, *begging, rescue*, *public benefit*, and *human trafficker *	*Proposal, exposure, fight, fund, attention, spread, criminal, donation *
Organizations/media accounts		*Shiqu Chen, ministry of public security, police, nationwide, power, action, attention, and society *
Grassroots stars		*Charles Xue, netizen, photograph, forward, love, charity, mother, and home *
Ordinary individuals		*Support, call the police, volunteer, hope, safe, family, struggle, and frenzied *

**Table 8:** Keywords distribution on the event of “Tianyi Li.”

Event 4: Tianyi Li		
Category	Cooccurrence Keywords	Keywords with Category Label
Celebrity	*Tianyi Li*, *rape by turns*, *suspect*, *police*, *Ge Meng*, and *Shuangjiang Li *	*Case, China, media, netizen, journalists, society, and educate *
Organizations/media accounts		*Lawyer, arrest, investigate, trial, victim, testify, and responsibility *
Grassroots stars		*Law, uncultured, bully, car, BMW, exposure, and labor camp *
Ordinary individuals		*Children, crime, parents, motherhood, tragedy, imperious, and pathetic *

**Table 9:** Keywords distribution on the event of “Xiaomi Phone.”

Event 5: Xiaomi Phone		
Category	Cooccurrence Keywords	Keywords with Category Label
Celebrity	*Xiaomi*, *phone*, *Jun Lei*, *company*, *release*, and *success *	*Internet, pioneer, fans, intellect, operator, market, and marketing *
Organizations/media accounts		*Domestic, release, configuration, users, forum, reserve, and official website *
Grassroots stars		*MIUI, Android, iPhone, Samsung, Meizu, hardware, experience, price, and battery *
Ordinary individuals		*Purchase, activity, sharing, enthusiastic, support, lottery, buying spree, and expect *

**Table 10:** Keywords distribution on the event of “Data Mining.”

Event 6: Data Mining		
Category	Cooccurrence Keywords	Keywords with Category Label
Celebrity	*Data Mining*, *analyzing*, *recommendation*, *Internet*, *user*, *information*, and *technology *	*Algorithm, research, machine learning, statistics, system, intelligent, prediction, and business *
Organizations/media accounts		*Recruitment, company, customer, marketing, product, engineer, and value *
Grassroots stars		*MicroBlog, advertising, precision, model, database, social, and application *
Ordinary individuals		*Sharing, learning, tools, search, develop, collect, and data *

**Table 11:** Keywords distribution on the event of “Gangnam Style.”

Event 7: Gangnam Style		
Category	Cooccurrence Keywords	Keywords with Category Label
Celebrity	*Gangnam Style*, *PSY*, *Korea*, *forward, and fashion *	*Video, ranking list, top spot, rap, popular, and culture *
Organizations/media accounts		*Internet, download, click, billion, wonderful, global, and hot *
Grassroots stars		*Kuda Kepang, imitate, MV, spoof, creative, mighty, and divine song *
Ordinary individuals		*Love, sharing, bravo, recommend, applaud, support, and circusee *

**Table 12:** Keywords distribution on the event of “Spring Festival.”

Event 8: Spring Festival		
Category	Cooccurrence Keywords	Keywords with Category Label
Celebrity	*Spring Festival*, *new year*, *red envelope*, *greetings*, *joyous*, and *Year of the Snake *	*Blessing, friends, parents, healthy, support, gift, and gratitude *
Organizations/media accounts		*Chinese, spring transportation, custom, New Year's Eve, safe, and firecracker *
Grassroots stars		*Temple fairs, lantern, play cards, party, traveling, and drinking *
Ordinary individuals		*Home, family reunion, Spring Festival Gala, lucky money, Valentine's Day, rose, and bell *

**Table 13:** The number of URLs adopted by different categories of users.

Category	Earthquake	College EE	Crack Down	Tianyi Li	Xiaomi Phone	Data Mining	Gangnam Style	Spring Festival
Celebrity	2774	2383	149	661	4009	1550	6477	1970
Organizations/media	5462	4648	137	781	9687	4930	11330	2744
Grassroots stars	2223	1340	40	213	4534	2139	9622	1776
Ordinary individuals	1603	1607	51	561	6325	1059	3402	3601
